# Population Based Outcomes of Cataract Surgery in Three Tribal Areas of Andhra Pradesh, India: Risk Factors for Poor Outcomes

**DOI:** 10.1371/journal.pone.0035701

**Published:** 2012-05-02

**Authors:** Rohit C. Khanna, Srinivasa Reddy Pallerla, Shiva Shankar Eeda, Bala Krishna Gudapati, Sandra D. Cassard, Padmaja Kumari Rani, Ghanshyam Palamaner Subash Shantha, Subhabrata Chakrabarti, Oliver D. Schein

**Affiliations:** 1 Brien Holden Eye Research Centre, L.V. Prasad Eye Institute, Banjara Hills, Hyderabad, India; 2 Allen Foster Research Centre for Community Eye Health, International Centre for Advancement of Rural Eyecare, L.V. Prasad Eye Institute, Hyderabad, India; 3 Andhra Pradesh Right to Sight Society, Hyderabad, India; 4 Wilmer Eye Institute, Johns Hopkins University, Baltimore, Maryland, United States of America; 5 Johns Hopkins Bloomberg School of Public Health, Baltimore, Maryland, United States of America; Medical University Graz, Austria

## Abstract

**Purpose:**

To report visual outcomes and risk factors for poor outcomes of cataract surgery in three Integrated Tribal Development Agency (ITDA) areas of Andhra Pradesh, India.

**Methods and Results:**

Using validated Rapid Assessment of Avoidable Blindness (RAAB) methodology, a population based cross-sectional study, was conducted in three ITDA areas. A two-stage sampling procedure was used to select 7281 participants aged 50 years and above. Vision assessment using a tumbling E chart and standard ocular examinations were completed. Visual outcomes and risk factors for poor outcomes were assessed among subjects undergoing cataract surgery (1548 eyes of 1124 subjects). Mean age at surgery was 67±8 years; Among the operated eyes, presenting visual acuity (PVA) and best corrected visual acuity (BCVA) worse than 6/18 was seen in 492 (31.8%; 95% CI, 29.5–34.2%) and 298 eyes (19.3%; 95% CI, 17.3–21.3%), respectively. Similarly, PVA and BCVA worse than 6/60 was seen in 219 (14.1%; 95% CI, 12.4–16%) and 147 eyes (9.5%; 95% CI, 8.1–11.1%), respectively. When either eye was taken into consideration, the PVA and BCVA worse than 6/18 was seen in 323 (20.1%; 95% CI, 18.9–23%) and 144 subjects (9.3%; 95% CI, 7.9–10.9%), respectively. PVA and BCVA worse than 6/60 was seen in 74 (4.8%; 95% CI, 3.8–6%) and 49 subjects (3.2%; 95% CI, 2.4–4.2%), respectively. Posterior capsular opacification was seen in 51 of 1316 pseudophakic eyes (3.9%; 95% CI, 2.9–5.1%). In multivariable analysis among pseudophakic subjects with PVA worse than 6/18, increasing age (p = 0.002) and undergoing free surgery (p = 0.05) were independent risk factors. Undergoing surgery before 2005 (p = 0.05) and being illiterate (p = 0.05) were independent risk factors for BCVA worse than 6/18.

**Conclusions:**

There are changing trends with improved outcomes in cataract surgery among these tribal populations of India. However, post-operative refractive error correction remains an issue, especially for those undergoing free surgeries.

## Introduction

Population based surveys in India in the past have identified cataract as the leading cause of blindness and visual impairment (VI) [1], [2]. The Government of India launched a World Bank-aided project in 1994 to eliminate cataract blindness in 7 states of the country with the highest prevalence of cataract related blindness and VI [2], [3]. As a result of this program, 15.3 million cataract surgeries were performed compared to a projected 11 million [3]. However, there were several concerns with respect to the quality of cataract surgeries performed under this program. Cataract surgery outcomes across different parts of the country varied significantly, with poor visual outcomes ranging between 11.8%–44.1% [4], [5], [6], [7], [8], [9], [10].

India has one of the largest tribal population in the world. The Government of India defined a tribal region based on certain characteristics [11], which include (and are not limited to) economically backward communities living in a primitive condition, having a distinct culture and usually living in isolation away from the mainstream. To the best of our knowledge, there are no reports on eye care and its implications from the tribal regions of India. This could be attributed to the poor accessibility of their regions of habitat [11]. The government has established the Integrated Tribal Development Agency (ITDA) to bring an overall development in these under-served areas. In the state of Andhra Pradesh (AP) in Southern India, the government received ∼1.5 million USD for Tribal Eye Care Project from the Department for International Development (DFID), UK, for a period of 3 years.

As there was no prior evidence on avoidable blindness from any tribal population in India, we conducted a Rapid Assessment of Avoidable Blindness (RAAB) survey in 3 tribal-inhabited areas of Andhra Pradesh (AP) to assess the prevalence of visual impairment and blindness in these regions. The objective of this paper is to report the visual outcomes of cataract surgery and also assess the risk factors contributing to poor outcome in these 3 ITDA areas.

## Methods

### Geographical distribution

The 3 ITDA selected for this study in Andhra Pradesh were Bhadrachalam in the Khammam district and Eturunagaram in the Warangal district (Area 1), Kota Rama Chandrapuram (K R Puram) in the West Godavari district and Rampa Chodavaram (R C Varam) in the East Godavari district (Area 2) and Srisailam, which is spread over 6 districts of Mahabubnagar, Nalagonda, Ranga Reddy, Kurnool, Guntur and Prakasam (Area 3). As per the 2001 census, the population of area 1 was 897,611, area 2 was 421,000 and area 3 was 554,439 [12].

**Table 1:** Comparison of characteristics of 1548 eyes based on their lens status.

Parameters	Aphakia–n (%)	Pseudophakia– n (%)	P value	Total – n (%)
Total eyes	232 (15)	1316 (85)		1548 (100)
Area 1	111 (47.8)	489 (37.2)		600 (38.8)
Area 2	33 (14.2)	283 (21.5)	0.018	316 (20.4)
Area 3	88 (37.9)	544 (41.3)		632 (40.8)
Proportion in tribals	46 (19.8)	306 (23.3)	0.34	352 (22.7)
Proportion in literate	8 (3.5)	125 (9.5)	0.004	133 (8.6)
Time of surgery				
≤1998	70 (30.2)	27 (2.1)		97 (6.3)
1999–2004	127 (54.7)	339 (25.8)	<0.001	466 (30.1)
2005–2009	35 (15.1)	950 (72.7)		985 (63.6)
Government sector operated	124 (53.4)	592 (45)	0.03	716 (46.3)
Proportion operated free	157 (67.7)	1099 (83.5)	<0.001	1256 (81.1)
V/A<6/60 (PVA)	116 (50)	103 (7.8)	<0.001	219 (14.2)
V/A<6/18 (PVA)	166 (71.6)	326 (24.8)	<0.001	492 (31.8)
V/A <6/60 (BVA)	74 (31.9)	73 (5.6)	<0.001	147 (9.5)
V/A <6/18 (BVA)	128 (55.2)	128 (55.2)	<0.001	298 (19.3)

Abbreviations: PVA: Presenting visual acuity; BVA: Best corrected visual acuity.

### Sample selection

A population based cross-sectional survey was conducted using validated RAAB methodology among subjects aged 50 years or older [13]. A two-stage sampling strategy involved Probability Proportionate to Size Sampling (PPSS) strategy for the selection of clusters (villages) followed by Compact Segment Sampling (CSS) for selection of households within the clusters [14], [15]. The population sizes in these villages were obtained from 2001 census data and an annual growth rate of 1.3% was added to obtain an estimate for 2009. For sample size calculations, the prevalence of blindness in each area was estimated to be 6%. It was estimated that 13% of the population would be 50 years or older [12]. Allowing for a 95% confidence interval, a precision of 20% (lower bound of 4.8%), design effect of 1.5 for cluster of 50 subjects and 10% non-response rate, the sample size required in each area was 2500.

### Study definitions

Blindness was defined as VA worse than 6/60 in the better eye with available correction and VI was defined as VA worse than 6/18 but not worse than 6/60 in the better eye with available correction [16].

### Definitions of ocular pathologies

Refractive error was defined as VA <6/18 improving ≥6/18 with pin hole. Cataract was defined as visible opacity in pupillary area impairing vision and partly or complete obscuration of red reflex on distant direct ophthalmoscopy. Glaucoma was defined as any 2 of the following 4 signs i) Neuroretinal rim reduced to < = 0.1 of cup disc (C:D) size ii) C:D ratio > = 0.8 iii) asymmetry of the cups > = 0.2 iv) any surgical/laser procedure performed for glaucoma and any fundus pathology other than glaucoma was characterized as posterior segment pathology.

The standard RAAB survey form was used (Annexure S1). Additional information was collected on the type of population residing in these areas (tribal or non-tribal) and literacy. Illiteracy was defined as not able to read or write simple text and numbers [17]. The study was approved by the Institutional Review Board of L V Prasad Eye Institute and conformed to the tenets of the Declaration of Helsinki. All subject aged 50 years and above in the population, in the research area, residing in the village at least for the previous 6 months from the initiation of the study and willing to give informed consent were enrolled. A written informed consent was obtained from all the subjects having a minimum level of literacy. However, subjects who did not have a formal education provided their consent based on a thumb impression in the consent form. All the study procedures was explained in detail to each subject by the study investigator, in presence of two to three community heads of the village.

### Training

Training for all the team members was given at L V Prasad Eye Institute, Hyderabad and an Inter Observer Variation Test (IOVT) was performed for each of these teams for the measurement of visual acuity (VA) and lens examination and to ensure acceptable agreement (Kappa value ≥0.6). Additionally, causes of blindness and VI were assessed by qualified ophthalmologists based on clinical examinations. For quality control measures, the clinical findings of all subjects with presenting visual acuity (PVA) <6/18 in either eye, those with previous cataract surgery and 10% of normal subjects in 6 preselected clusters (2 in each area), were further assessed by a second ophthalmic assistant and an ophthalmologist. Prior to undertaking the main study, a pilot study was conducted to ensure the standardization of all protocols.

**Table 2:** Proportion of eyes with pseudophakia and aphakia having presenting and best corrected visual acuity <6/18.

Parameters	Pseudophakic eyes		Aphakic eye	
	(n = 1316)		(n = 232)	
	PVA<6/18	BCVA <6/18	PVA <6/18	BCVA <6/18
	n (%)	n (%)	n (%)	n (%)
Total	326 (24.8)	170 (12.9)	166 (71.6)	128 (55.2)
50–59 years	30 (14.6)	16 (7.8)	14 (66.7)	13 (61.9)
60–69 years	154 (25.9)	80 (13.5)	58 (73.4)	46 (58.2)
≥70 years	142 (27.5)	74 (14.3)	94 (71.2)	69 (52.3)
Male	134 (23.2)	71 (12.3)	62 (72.1)	51 (59.3)
Female	192 (26.0)	99 (13.4)	104 (71.2)	77 (52.7)
Tribal	83 (27.1)	41 (13.4)	31 (67.4)	26 (56.5)
Non tribal	243 (24.1)	129 (12.8)	135 (72.6)	102 (54.8)
Literate	19 (15.2)	8 (6.4)	4 (50)	4 (50)
Illiterate	307 (25.8)	162 (13.6)	162 (72.3)	124 (55.4)
Glasses				
Yes	60 (20.9)		78 (57.4)	
No	266 (25.9)		88 (91.7)	
2005–2009	221 (23.3)	108 (11.4)	25 (71.4)	20 (57.1)
1999–2004	99 (29.2)	57 (16.8)	92 (72.4)	75 (59.1)
<1999	6 (22.2)	5 (18.5)	49 (70)	33 (47.1)
Paid	37 (17.1)	19 (8.8)	48 (64)	33 (44)
Free	289 (26.3)	151 (13.7)	118 (75.2)	95 (60.5)
NGO/Private	156 (21.6)	84 (11.6)	72 (66.7)	53 (49.1)
Government	170 (28.7)	86 (14.5)	94 (75.8)	75 (60.5)
Area 1	112 (22.9)	52 (10.6)	73 (65.8)	51 (46)
Area 2	72 (25.4)	46 (16.3)	26 (78.8)	21 (63.6)
Area 3	142 (26.1)	72 (13.2)	67 (76.1)	56 (63.6)

PVA: Presenting Visual Acuity; BCVA: Best Corrected Visual Acuity; NGO: Non-governmental Organization.

### Data collection procedures

The epidemiological data were collected from the last week of July to September 2009. A local health worker in each cluster helped with enumeration as well as verification of age and the examination procedure was explained to the subjects. Visual acuity was measured in daylight conditions using the tumbled ′E′ charts optotype 6/60 on one side and 6/18 on other side. If the subject's VA was worse than 6/18, a pinhole test was performed. For aphakic subjects, a+10D lens or their available correction was used along with a pinhole correction. Pinhole measured visual acuity was used as a surrogate for best corrected visual acuity (BCVA). If the subject's VA did not improve with pinhole correction, the subject was examined by an ophthalmologist using a portable uni-ocular slit lamp for the anterior segment. For assessment of lens status, torch and distant direct ophthalmoscope were used in a shaded or semi-dark environment without pupillary dilatation. Then 1% Tropicamide drops were instilled and a fundus examination was done to check for any posterior segment pathology. The principal cause of blindness or VI was recorded by the ophthalmologist and if there was more than one cause, the most treatable cause was recorded. If the subject required any treatment, he was referred to the nearest secondary or tertiary eye care facility with a referral slip.

**Table 3:** Cause of Visual impairment and blindness stratified by category of visual impairment and blindness.

Causes	<6/18–6/60	<6/60	Total
	n (%)	n (%)	
Refractive error	174 (63.7)	17 (7.8)	191 (38.8)
Aphakia (uncorrected)	10 (3.7)	64 (29.2)	74 (15)
Surgical complications	31 (11.4)	47 (21.5)	78 (15.9)
Other corneal scars	2 (0.7)	*9 (4.1)	11 (2.2)
Glaucoma	4 (1.5)	13 (5.9)	17 (3.5)
Other posterior segment	52 (19.1)	69 (31.5)	121 (24.6)
Overall n (%)	273 (100)	219 (100)	492 (100)

* Include one eye with phthisis.

Subjects who had undergone cataract surgery were asked about the date and place of their surgery and whether they paid for the services or if it was free. If the subject was absent, two revisits were made, before classifying the subject as unavailable.

Data were collected and entered on the same day, independently by two data entry operators (Annexure S1). Both the databases were checked for consistency using the RAAB software.

### Data Analysis

Data analysis was performed with Stata software (version 11.0) [18]. Continuous variables were compared using Student's t test or one-way analysis of variance (ANOVA), while categorical variables were analyzed using chi-square test. Risk factors for VA worse than 6/18 were analysed by multiple logistic regression with generalized estimating equation (GEE) along with robust variance estimation to account for the correlation between both the eyes in an individual [19], [20]. Multi-collinearity between variables was assessed looking at the variance inflation factor and fitness of the model was assessed using Hosmer Lemeshow test for goodness of fit [21]. Since the two surgical procedures for aphakia and pseudophakia are quite different, a separate logistic regression was performed for each of them.

**Table 4:** Logistic regression showing risk factor for blindness and visual impairment for presenting visual acuity (PVA) and best corrected visual acuity (BCVA) in pseudophakic eyes.

	For PVA <6/18 in pseudophakic eyes				For BCVA <6/18 in pseudophakic eyes			
	n = 1316 eyes				n = 1316 eyes			
	*OR	P value	#OR	P value	*OR	P value	#OR	P value
Age								
50–59	Ref							
60–69	2.05	0.001	1.98	0.002	1.85	0.03	1.64	0.1
≥70	2.23	<0.001	2.19	0.001	1.99	0.02	1.80	0.05
Overall		0.001		0.002		0.05		0.14
Gender								
Male	Ref							
Female	1.17	0.27	1.16	0.31	1.11	0.57	1.08	0.7
Tribal								
Yes	Ref							
No	0.85	0.32	0.89	0.53	0.95	0.79	1.13	0.57
Literacy								
Literate	Ref							
Illiterate	1.94	0.01	1.63	0.09	2.30	0.03	2.16	0.05
Glasses								
Yes	Ref							
No	1.32	0.12	1.26	0.19				
Time								
2005–09	Ref							
1999–04	1.36	0.04	1.31	0.08	1.58	0.01	1.35	0.03
<1999	0.94	0.9	1.03	0.95	1.77	0.27	2.09	0.16
Overall		0.11		0.21		0.03		0.05
Paying								
Paid	Ref							
Free	1.74	0.005	1.52	0.05	1.66	0.046	1.57	0.12
Place								
NGO/PVT	Ref							
GVT.	1.47	0.004	1.31	0.09	1.29	0.13	1.23	0.33
Area								
Area 1	Ref							
Area 2	1.15	0.46	1.12	0.58	1.63	0.04	1.77	0.03
Area 3	1.19	0.43	1.20	0.27	1.28	0.22	1.34	0.16
Overall		0.52		0.53		0.11		0.08

PVA: Presenting Visual Acuity; BCVA: Best Corrected Visual Acuity; OR: Odds Ratio; CI: Confidence Interval; NGO: Non-governmental Organization; H-L: Hosmer-Lemeshow goodness of fit; GVT: government; NGO: non-governmental organization; PVT: private sector; *: indicates odds ratios of uni-variate analysis, #: indicates odds ratios of multi-variate analysis.

## Results

During the study period, 7500 subjects were enumerated in three tribal zones and 7281 (97.1%) were examined. 1124 subjects had undergone cataract surgery, yielding an overall prevalence of 15.4% (95% CI, 14.6–16.3%). Of them, 424 subjects (37.7%) had undergone bilateral surgery. A higher proportion of surgeries were performed in the non-tribal population compared to the tribal population (19.6% versus 9%; p<0.001) and among illiterates as compared to literates (16% versus 11%; p<0.001).

Overall, the mean ages of the subjects who underwent cataract surgery was 67±8 years (median: 65 years and range: 50–90 years). The mean age of the female subjects (n = 642) was lower than males at the time of surgery (p = 0.001). Only 25.4% of the operated subjects were using glasses. Of the unilaterally operated eyes, 59 had aphakia and 641 had pseudophakia, while among the bilaterally operated subjects, 52 had aphakia in both eyes and 303 had pseudophakia in both the eyes. The remaining 69 subjects had aphakia in one eye and pseudophakia in the other eye. Thus, despite 232 (15%) eyes being aphakic, 90.1% subjects had pseudophakia in at least one eye.

The mean time since surgery was 4.4±4.5 years (median = 3 years). Among the different settings that were availed for surgery, 708 (45.7%) eyes were operated in a government hospital, 569 (36.8%) eyes in the non-governmental organizations, 263 (17%) eyes in the private sector and the remaining 8 (0.5%) in eye camps. Since the 8 eyes operated in camps were performed in the government sector, they were grouped with those operated in the government sector for further analysis. For comparisons across different studies, those operated in the private sector were grouped to those operated in the non-governmental organizations. A total of 1256 (81.1%) eyes were operated for free.

Table 1 provides a comparison of different characteristics of the 1548 eyes based on their lens status (aphakia and pseudophakia). As seen in this table, most of the aphakia surgeries were done before 2005 (p<0.001) and a relatively higher proportion of these were operated in the government sector (p = 0.03) and among the illiterate population (p = 0.004).

### Visual impairment and blindness

As seen in table 1, PVA worse than 6/60 and worse than 6/18 was found in 219 (14.2%; 95% CI, 12.4–16%) and 492 eyes (31.8%; 95% CI, 29.5–34.2%), respectively. The BCVA worse than 6/60 and worse than 6/18 was found in 147 (9.5%; 95% CI, 8.1–11.1%) and 298 eyes (19.3%; 95% CI, 17.3–21.3%), respectively. When either eye was taken into consideration, the PVA and BCVA worse than 6/18 was seen in 323 (20.1%; 95% CI, 18.9–23%) and 144 subjects (9.3%; 95% CI, 7.9–10.9%), respectively. PVA and BCVA worse than 6/60 was seen in 74 (4.8%; 95% CI, 3.8–6%) and 49 subjects (3.2%; 95% CI, 2.4–4.2%), respectively. PVA and BCVA worse than 6/18 and worse than 6/60 was significantly more prevalent among the aphakic compared to pseudophakic eyes (p<0.001). Table 2 shows the proportion of eyes with pseudophakia and aphakia having PVA and BCVA worse than 6/18 stratified by various risk factors. There was no difference in the proportion of visual impairment (p = 0.7) or blindness (p = 0.24) in the 3 areas.

### Causes of visual impairment and blindness

Overall, refractive error, uncorrected aphakia, surgical complications and posterior segment disorders were the major causes of VI and blindness (Table 3). Among the subjects with VA worse than 6/60, uncorrected aphakia, surgical complications and posterior segment disorders were relatively more common, while for those with VA worse than 6/18 but better than 6/60, uncorrected refractive error was the common cause of VI and blindness. Posterior capsular opacification was seen in 51 of 1316 pseudophakic eyes (3.9%; 95% CI, 2.9–5.1%)

### Risk factors for blindness and VI

Table 4 and Table S1 exhibits the risk factor for blindness and VI for PVA and BCVA in pseudophakic eyes, wherein, for PVA, increasing age (p = 0.002) and availing free surgery (p = 0.05) were an independent risk factors, illiteracy (p = 0.09) and surgeries performed at the government sector (p = 0.09) were not the major risk factors. Similarly, for BCVA, illiteracy (p = 0.05) and a history of cataract surgery before year 2005 (p = 0.05) were marginal risk factors for blindness and VI. Among the aphakic subjects, abstaining from the use of glasses was the only independent risk factor (p<0.001) for PVA (data not shown). It may be noted here that the power to detect significant risk factors in aphakic subjects were limited due to vagaries of small sample size.

## Discussion

The overall prevalence of cataract surgery was much higher than that reported from India a decade ago and is relatively higher compared to neighboring and some developed countries [5], [6], [7], [8], [22], [23], [24], [25], [26], [27], [28]. Our estimates are similar to the prevalence seen in the urban cohort (15.7%) but lower than the rural cohort (21.9%) of Chennai Glaucoma Study (CGS) and also lower than that reported from Navsari in Gujarat (17.6%) [9], [10].

Among the 1548 eyes, 15% were aphakics, which was much lower than that reported a decade ago [5], [6], [7], [8]. The overall prevalence of aphakia was comparable to reports from Navsari in Gujarat (15.9%)[10], but was lower than the urban and rural cohorts of CGS (27.2% and 44.5%, respectively) [9], [10]. The proportion of intraocular lens implantation was much higher that reported a decade earlier from India [5], [6], [7], [8]. These temporal trends suggest that most of the surgeons have passed the transition phase of conversion from non-IOL surgery to IOL surgery.

PVA and BCVA > = 6/18 were seen in 68.2% and 80.7% of the eyes, respectively and there was no significant difference in the 3 tribal areas. This was much higher than that reported from a recent study from Navsari in Gujarat (PVA and BCVA ≥6/18 seen in 50.7% and 74.5%, respectively)[10], CGS (PVA and BCVA ≥6/18 seen in 54.2% and 78.4% respectively)[9] and Bharatpur in Rajasthan (PVA and BCVA ≥6/18 seen in 31.5% and 58.8% respectively) [7]. Though the PVA was better than Tirunelveli and Sivaganga (PVA ≥6/18 seen in 64% and 60.3% respectively), the BCVA was comparable to both the areas (BCVA ≥6/18 seen in 83% and 84.5% respectively) [6], [8]. It was also comparable to Lumbini zone and Chitwan district of Nepal [24], but better than Satkhira district in Bangladesh [28]. The difference in PVA and BCVA reported from other parts of India and neighboring countries could be due to the difference in proportion of pseudophakics in these populations as well as the use of IOL with appropriate power.

Based on PVA, only 14.2% eyes were blind and the proportion was significantly higher for aphakics than pseudophakics. This was lower to that observed at Navsari in Gujarat (18%) [10], and the urban and rural cohorts of CGS (17.2% and 26.8%, respectively) [9]. This was also comparable to Tirunelveli and Sivaganga (11.8% and 13.8% respectively) [6], [8]; and much lower than Bharatpur in Rajasthan (44.1%) [7] and the urban and rural cohorts in the previously conducted Andhra Pradesh Eye Disease Study (APEDS) (21.4% and 24.5% respectively) [4], [5]. Similar variation in proportions of blindness were reported from the neighboring regions of Lumbini zone and Chitwan district in Nepal (13.8%) [24], Ratuhat district in Nepal (20%) [27], Satkhira district in Bangladesh (23.5%) [28], Liwan in China (15.6%) [23] and Hong Kong (11.2%) [25]. It may be noted that the proportion of blindness in the population was highly influenced by the blindness in aphakics in the present study. Thus, the proportion of aphakics in other populations may also explain the differences in the prevalences of blindness among them.

Based on PVA, the proportion of blindness in pseudophakic population was only 7.8%, which is similar to that reported from Bharatpur in Rajasthan (14%) [7], CGS(11.7%) [9], Tirunelveli (5.1%) [8], Sivaganga (4.2%) [6] and APEDS (5.6%) [5]. It was also comparable to the neighboring regions of Lumbini zone and Chitwan district of Nepal (7.9%) [24], Ratuhat district in Nepal (10%) [27], Satkhira district in Bangladesh (6%) [28] and Hong Kong (7.8%) [25]. It may be stated here that as the prevalence of aphakia in a population decreases, the proportion of post-cataract surgery blindness also reduces simultaneously.

The differences in PVA and BCVA underscores the importance of adequate post-operative refraction and use of spectacles. The use of spectacles was only 25.4%, which was much lower than Navsari in Gujarat (54.2%) [10], Tirunelveli (35%) [8], Bharatpur in Rajasthan (56%) [7] and Sivaganga (48.6%) [6]. As stated earlier, this could be due to the increased proportion of pseudophakics in this population. In the pseudophakic population of Tirunelveli and Sivaganga the use of spectacles was only ranging from 10–20% [6], [8]. The possible reason for low use of spectacles in the pseudophakic population could be that these subjects may not feel the need for use of spectacles as they are able to carry on their daily activities without them. Another possible reason could be due to the issues related to accessibility and affordability of refractive error services. The use of spectacles in pseudophakics was also much lower than the developed regions of Hong Kong (40%) [25] and Latinos in Los Angeles (70%) [22].

The major cause of VI was uncorrected refractive error and for blindness was uncorrected aphakia, surgical complications and posterior segment disorders. This was similar to the other studies from India [4], [5], [6], [7], [8], [9], [10]. This underscores the importance of rigorous pre-operative comprehensive examination to rule out any pre-existing pathology and adequate training to manage surgical complications. Posterior capsular opacification (PCO) was seen in only 3.9% of pseudophakic subjects and was comparable to Tiruneveli (5.3%) [8] and Sivaganga (2.7%) [6] and higher than reportd in Nepal (0.5%) [27]. However, it was lower than Navsari in Gujarat (16.5%) [10] and CGS (12.5%) [9]. It was also lower than Lumbini zone and Chitwan district of Nepal (8%) [24].

Analysis of risk factors for VA<6/18 in pseudophakic eyes for PVA and BCVA suggest that there are issues related to refractive error services, especially for those who were operated for free. As the use of glasses did not differ between those operated for free and the paid subjects (21.2% versus 24.9%; p = 0.23), this could be attributed to an incorrect prescription in these subjects. As far as the National Program for Control for Blindness (NPCB) policy is concerned, there is only one time funding provided for prescribing spectacles to the post-operative subjects. As a usual practice, most of these refractions are done still in camp settings in the rural areas where mass refractions are done in a single day. It is possible that there might be more errors in refractions done in these make shift units. Apart from this, the refractive error status of pseudophakic subjects change over a period of time. However, due to issues related to affordability and accessibility of refractive error services, these subjects are very unlikely to go for repeat refraction or for purchase of a new pair of glasses. Poor outcome for BCVA in subjects with an early surgical intervention could be due to other ocular co-morbidity in them. Illiteracy was used as proxy for socio-economic status and it is possible that various other factors associated with illiteracy or poverty might be responsible for poor outcomes in these subjects.

Despite our best efforts, there were some limitations in this study. As visual fields were not performed on these subjects, we might have missed some VI or blindness due to visual field defects. We also did not assess the type of cataract surgery performed on these subjects as well as the details of surgical complications.

In summary, compared to previous studies done a decade earlier, there was an overall changing trend related to cataract surgery outcomes seen in this population. Though we did not have a baseline data before the launch of the Tribal Eye Care project, it is possible that these results might reflect the success of the Tribal Eye Care Project launched by the government in these remote areas Andhra Pradesh. This study has also shown that there were some issues related to the use of spectacles and visual outcomes in those operated free. Proper post-operative refractions, adequate follow-up and provision of glasses might help to comprehensively address this issue.

## Supporting Information

Table S1 Logistic regression showing risk factor for blindness and visual impairment for presenting visual acuity (PVA) and best corrected visual acuity (BCVA) in pseudophakic eyes (with 95% CI).
(DOC)

Annexure S1 Data entry form.
(DOC)
