# Supplementary material for: Population Based Outcomes of Cataract Surgery in Three Tribal Areas of Andhra Pradesh, India: Risk Factors for Poor Outcomes
Source: PLoS One. 2012 May 2;7(5):e35701. doi: 10.1371/journal.pone.0035701 (PMC3342298; doi:10.1371/journal.pone.0035701)
Supplement: Table S1 — Logistic regression showing risk factor for blindness and visual impairment for presenting visual acuity (PVA) and best corrected visual acuity (BCVA) in pseudophakic eyes (with 95% CI). (DOC) [file pone.0035701.s001.doc]

**Table S1: Logistic regression showing risk factor for blindness and visual impairment for presenting visual acuity (PVA) and best corrected visual acuity (BCVA) in pseudophakic eyes**

|  | For PVA < 6/18 in pseudophakic eyes | | | | For BCVA < 6/18 in pseudophakic eyes | | | |
| --- | --- | --- | --- | --- | --- | --- | --- | --- |
|  | n = 1316 eyes | | | | n = 1316 eyes | | | |
|  | *OR | P value | #OR | P value | *OR | P value | #OR | P value |
|  | (95% CI) |  | (95% CI) |  | (95% CI) |  | (95% CI) |  |
| Age |  |  |  |  |  |  |  |  |
| 50 - 59 | Ref |  |  |  |  |  |  |  |
| 60 - 69 | 2.05 | 0.001 | 1.98 | 0.002 | 1.85 | 0.03 | 1.64 | 0.1 |
|  | 1.33-3.16 |  | 1.28-3.05 |  | 1.05-3.26 |  | 0.92-2.94 |  |
| ≥ 70 | 2.23 | <0.001 | 2.19 | 0.001 | 1.99 | 0.02 | 1.80 | 0.05 |
|  | 1.44-3.45 |  | 1.41-3.42 |  | 1.12-3.53 |  | 1.0 – 3.23 |  |
| Overall |  | 0.001 |  | 0.002 |  | 0.05 |  | 0.14 |
| Gender |  |  |  |  |  |  |  |  |
| Male | Ref |  |  |  |  |  |  |  |
| Female | 1.17 | 0.27 | 1.16 | 0.31 | 1.11 | 0.57 | 1.08 | 0.7 |
|  | 0.89-1.53 |  | 0.87-1.56 |  | 0.78-1.56 |  | 0.75-1.55 |  |
| Tribal |  |  |  |  |  |  |  |  |
| Yes | Ref |  |  |  |  |  |  |  |
| No | 0.85 | 0.32 | 0.89 | 0.53 | 0.95 | 0.79 | 1.13 | 0.57 |
|  | 0.62-1.17 |  | 0.62-1.28 |  | 0.63-1.41 |  | 0.74-1.73 |  |
| Literacy |  |  |  |  |  |  |  |  |
| Literate | Ref |  |  |  |  |  |  |  |
| illiterate | 1.94 | 0.01 | 1.63 | 0.09 | 2.30 | 0.03 | 2.16 | 0.05 |
|  | 1.16-3.25 |  | 0.94-2.83 |  | 1.09-4.85 |  | 0.98-4.75 |  |
| Glasses |  |  |  |  |  |  |  |  |
| Yes | Ref |  |  |  |  |  |  |  |
| No | 1.32 | 0.12 | 1.26 | 0.19 |  |  |  |  |
|  | 0.93-1.86 |  | 0.89-1.79 |  |  |  |  |  |
| Time |  |  |  |  |  |  |  |  |
| 2005 - 09 | Ref |  |  |  |  |  |  |  |
| 1999 - 04 | 1.36 | 0.04 | 1.31 | 0.08 | 1.58 | 0.01 | 1.35 | 0.03 |
|  | 1.02-1.8 |  | 0.97-1.77 |  | 1.1-2.25 |  | 1.04-2.18 |  |
| < 1999 | 0.94 | 0.9 | 1.03 | 0.95 | 1.77 | 0.27 | 2.09 | 0.16 |
|  | 0.38-2.33 |  | 0.42-2.53 |  | 0.64-4.9 |  | 0.75-5.79 |  |
| Overall |  | 0.11 |  | 0.21 |  | 0.03 |  | 0.05 |
| Paying |  |  |  |  |  |  |  |  |
| Paid | Ref |  |  |  |  |  |  |  |
| Free | 1.74 | 0.005 | 1.52 | 0.05 | 1.66 | 0.046 | 1.57 | 0.12 |
|  | 1.19-2.54 |  | 0.99-2.33 |  | 1.0-2.73 |  | 0.9-2.7 |  |
| Place |  |  |  |  |  |  |  |  |
| NGO/PVT | Ref |  |  |  |  |  |  |  |
| GVT. | 1.47 | 0.004 | 1.31 | 0.09 | 1.29 | 0.13 | 1.23 | 0.33 |
|  | 1.13-1.91 |  | 0.96-1.8 |  | 0.93-1.81 |  | 0.81-1.85 |  |
| Area |  |  |  |  |  |  |  |  |
| Area 1 | Ref |  |  |  |  |  |  |  |
| Area 2 | 1.15 | 0.46 | 1.12 | 0.58 | 1.63 | 0.04 | 1.77 | 0.03 |
|  | 0.8-1.66 |  | 0.75-1.68 |  | 1.04-2.57 |  | 1.07-2.92 |  |
| Area 3 | 1.19 | 0.43 | 1.20 | 0.27 | 1.28 | 0.22 | 1.34 | 0.16 |
|  | 0.88-1.61 |  | 0.87-1.64 |  | 0.86-1.91 |  | 0.89-2.0 |  |
| Overall |  | 0.52 |  | 0.53 |  | 0.11 |  | 0.08 |
| H-L fit |  |  | 8.39 | 0.4 |  |  | 6.72 | 0.57 |

PVA: Presenting Visual Acuity; BCVA: Best Corrected Visual Acuity; OR: Odds Ratio; CI: Confidence Interval; NGO: Non-governmental Organization; H-L: Hosmer-Lemeshow goodness of fit; GVT: government; NGO: non-governmental organization; PVT: private sector; *: indicates odds ratios of uni-variate analysis, #: indicates odds ratios of multi-variate analysis
