# Supplementary figures and images for: Population Based Outcomes of Cataract Surgery in Three Tribal Areas of Andhra Pradesh, India: Risk Factors for Poor Outcomes
Source: PLoS One. 2012 May 2;7(5):e35701. doi: 10.1371/journal.pone.0035701 (PMC3342298; doi:10.1371/journal.pone.0035701)

**Annexure S1: Data entry form**


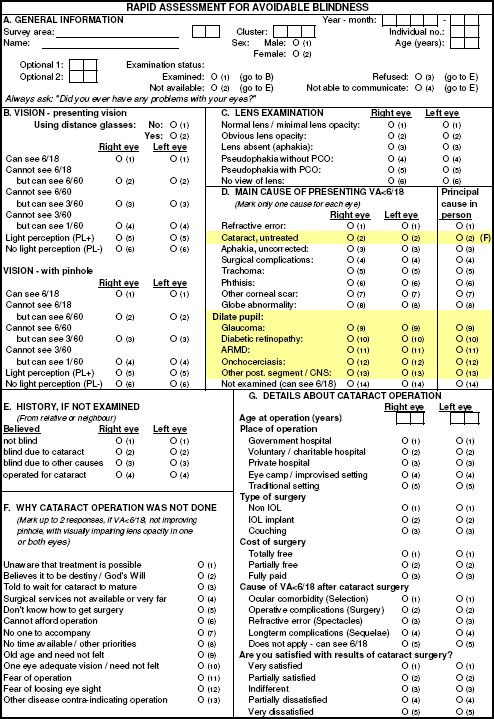

Supplement: Annexure S1 — Data entry form. (DOC) [file pone.0035701.s002.doc]
